# Diet quality and cardiometabolic health in childhood: the Generation R Study

**DOI:** 10.1007/s00394-021-02673-2

**Published:** 2021-09-15

**Authors:** Noreen Z. Siddiqui, Anh N. Nguyen, Susana Santos, Trudy Voortman

**Affiliations:** 1 Department of Epidemiology, Erasmus MC, University Medical Center, PO Box 2040, 3000 CA Rotterdam, The Netherlands; 2 The Generation R Study Group, Erasmus MC, University Medical Center, Rotterdam, The Netherlands; 3 Department of Pediatrics, Sophia Children’s Hospital, Erasmus MC, University Medical Center, Rotterdam, The Netherlands; 4 Division of Human Nutrition & Health, Wageningen University & Research, Wageningen, The Netherlands

**Keywords:** Children, Dietary patterns, Nutrition, Cardiometabolic health, Obesity, Metabolic syndrome

## Abstract

**Purpose:**

Diet is an important determinant of cardiometabolic disease risk in adults. We aimed to study associations of diet quality with cardiometabolic health in school-age children.

**Methods:**

This study was embedded in the Generation R Study a prospective population-based cohort in Rotterdam, the Netherlands and included 3991 children. Food intake was assessed with a Food-Frequency Questionnaire at age 8 years. A diet quality score (0–10) was calculated reflecting adherence to age-specific dietary guidelines. The following outcome variables were measured at age 10 years and used to create a continuous cardiometabolic risk factor score: body fat percentage, insulin, triglycerides, HDL cholesterol, and systolic and diastolic blood pressure. Outcomes were expressed in age- and sex-specific standard deviation scores (SDS). Multivariable linear regression models were used to assess associations between the diet quality score and the cardiometabolic risk factor score and with the individual cardiometabolic risk factors.

**Results:**

In models adjusted for socioeconomic and lifestyle factors and BMI, a higher diet quality was associated with a lower cardiometabolic risk factor score [− 0.08 per point higher diet score, (95% CI − 0.15, − 0.001)]. This association was mainly driven by associations of higher diet quality with lower systolic [− 0.04 SD (95% CI − 0.06, − 0.01)] and diastolic blood pressure [− 0.05 SD, (95% CI − 0.07, − 0.02)]. No statistically significant associations were found for insulin, triglycerides, HDL cholesterol, or body fat percentage as individual factors.

**Conclusions:**

We found an association between higher diet quality and better cardiometabolic health in childhood, mainly driven by a lower blood pressure. Further research is needed to explore associations of diet quality in childhood with long-term cardiometabolic health.

**Supplementary Information:**

The online version contains supplementary material available at 10.1007/s00394-021-02673-2.

## Introduction

The nutrition transition is a concept implemented by Popkin [1], who described the changes of population’s diets throughout the years. These dietary changes, especially the transition from traditional varied eating to a western diet, were suggested to result in poor health outcomes and could lead to cardiovascular diseases [2, 3], which is currently the number one leading cause of death globally [4]. Although these diseases usually do not occur until later adulthood, cardiometabolic risk factors, such as high blood pressure, can predict the risk of developing cardiovascular diseases [5]. These cardiometabolic risk factors can already be observed among children [2]. Children can for example already exhibit increased levels of triglycerides, insulin resistance, and hypertension [6, 7]. Eventually, worsening of cardiometabolic health during childhood leads to an increased risk of cardiovascular diseases later in life [2].

Cardiometabolic health is strongly influenced by lifestyle factors, including diet [8]. In adults, a healthy dietary pattern rich in fruit, vegetables, seeds, whole grains, legumes and soy products has been shown to be associated with a lower cardiometabolic disease risk [9]. However, evidence for whether diet is already associated with cardiometabolic health in childhood is inconsistent. It has been reported that polyunsaturated fatty acid intake was inversely correlated with low-density lipoprotein (LDL) cholesterol in children aged 1–4 years [10], whereas a diet rich in red meat, refined carbohydrates, and saturated fatty acids was associated with higher LDL cholesterol in children aged 11–18 years [11]. Because the human diet is based on multiple food products rather than one single food or nutrient and because multiple foods and nutrients have been linked to cardiometabolic health, using a dietary pattern approach, in which overall diet quality can be studied, has been recommended as method to study the relation between diet and cardiometabolic health [12]. We previously observed no associations between diet quality of 1-year-old children and their cardiometabolic health at the age of 6 years in the Generation R Study [5]. Because of these inconsistent findings and limited number of studies that examined associations of overall diet in children with cardiometabolic health [2], our aim was to examine associations of diet quality with cardiometabolic risk factors and overall cardiometabolic health in school-age children (Supplementary Fig. 1).

## Materials and methods

### Study design and study population

This study was embedded in the Generation R Study, a population-based prospective cohort from fetal life onward in Rotterdam, the Netherlands [13]. In total, 9778 pregnant women were enrolled with a delivery date between April 2002 and January 2006. The Generation R Study was approved by the Medical Ethics Committee of Erasmus University Medical Center. Written informed consent was provided by parents of all children who participated in the study [13].

**Fig. 1:**
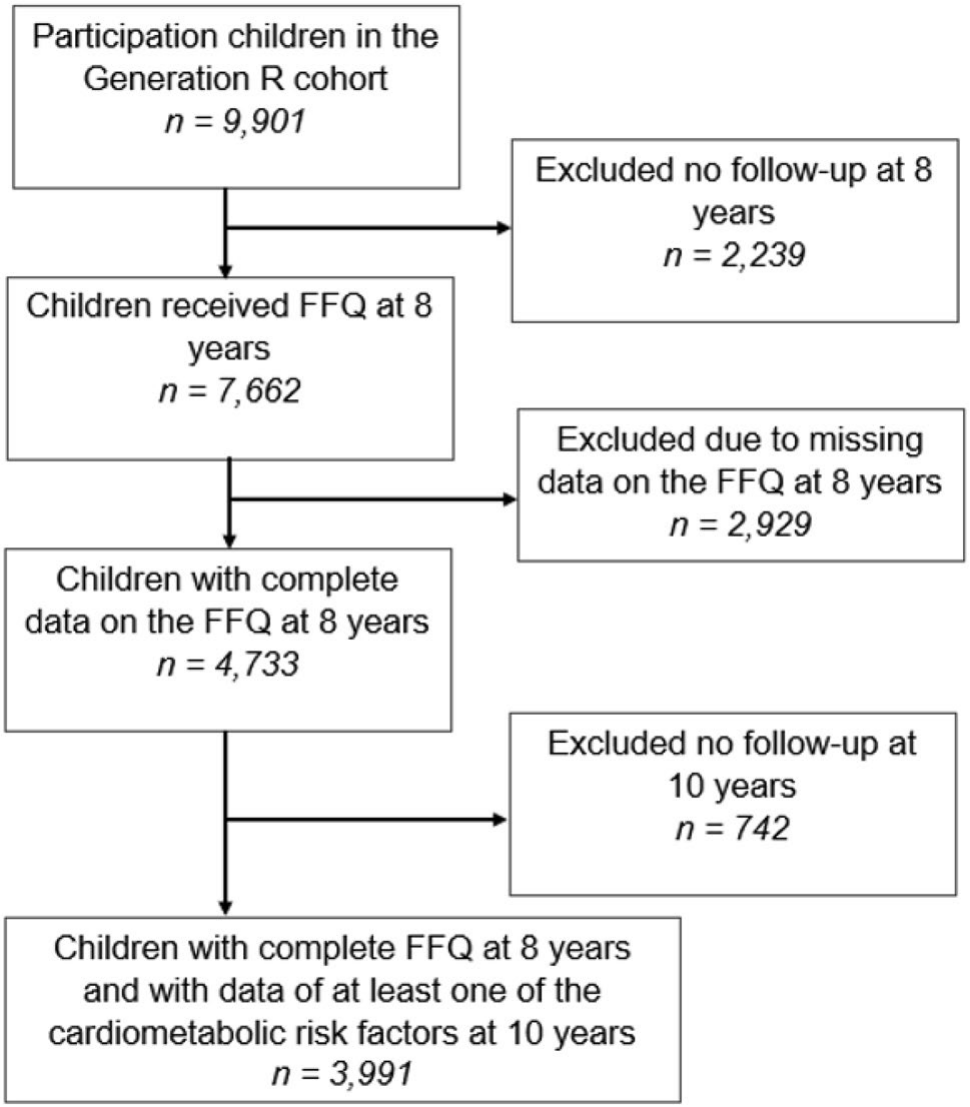
Flowchart study population

A total of 7662 mothers were invited to fill out a questionnaire on dietary intake of their child at the age of 8 years. We had complete dietary data for 4733 children. Of these children, 742 children were excluded because of no follow-up at age 10 years. Our study population comprises 3991 children with data available on dietary intake at 8 years and at least one of the cardiometabolic health outcomes at 10 years (Fig. 1).

### Dietary intake

We used a validated semi-quantitative Food-Frequency Questionnaire (FFQ) to assess child dietary intake at their median age of 8.1 (IQR 8.0–8.2) years. This FFQ included 71 items, that were selected based on the results from the National Food Consumption Survey among children in the Netherlands [12, 14]. Parents were asked to indicate how many times a week the children consumed each of the food items and subsequently to indicate average portion sizes, specified in natural units, household units or grams [14]. Additional questions were included about specific types or brands of foods for 27 items, and preparation methods. Energy and nutrient intakes were calculated using Dutch Food Composition Tables [12]. The FFQ has previously been validated in Dutch children for energy intake using the doubly labeled water method (Pearson’s *r* = 0.62) [14]. Further details of the FFQ are provided elsewhere [12, 14].

### Diet quality score

We used a diet quality score which was developed to indicate adherence to age-specific dietary guidelines [12]. The diet quality score consisted of ten components: fruit (≥ 150 g/day), vegetables (≥ 150 g/day), whole grains (≥ 90 g/day), fish (≥ 60 g/week), legumes (≥ 84 g/week), nuts (≥ 15 g/day), dairy (≥ 300 g/day), oils and soft or liquid margarines (≥ 30 g/day), sugar-containing beverages (SCB) (≤ 150 g/day), and high-fat and processed meat (≤ 250 g/week) [12]. For each component, the ratio of reported and recommended intake was calculated resulting in a continuous score with a minimum of 0 when the food items were not consumed and a maximum of 1 when the amount of the cut-off value or more was consumed [12]. These scores were inversed for SCB and high-fat and processed meat, with a higher score indicating a lower intake. Scores for the individual components were summed, resulting in a continuous score ranging from 0 to 10, with a higher score indicating a better diet quality. More details on this score are described elsewhere [12].

### Cardiometabolic risk factor score

The children were invited for a physical examination at the research center of the Erasmus MC-Sophia Children’s Hospital around their age of 10 years [median 9.7 (IQR 9.6–9.8)]. Body fat mass was measured with a Dual-energy X-ray Absorptiometry scanner (DXA) (iDXA scanner, GE Healthcare, Madison, WI, USA). We divided total body fat mass in kilograms by total weight in kilograms and multiplied the outcome by 100 to calculate body fat percentage (BF%). Serum concentrations of insulin, triglycerides, and high-density lipoprotein (HDL) cholesterol were determined in non-fasting blood samples [13, 15]. LDL cholesterol was calculated according to the Friedewald formula. Systolic and diastolic blood pressure (SBP and DBP) were measured using a sphygmomanometer (Datascope Accutorr Plus) [16]. Blood pressure was measured at the right brachial artery for four times with intervals of 1 min and the mean was calculated using the last three measurements of each participant [5, 16]. Age- and sex-specific SD scores (SDS) were calculated for all individual outcome variables. Subsequently, we calculated a continuous cardiometabolic risk factor score, according to the following formula: BF% SDS + 0.5 × SBP SDS + 0.5 × DBP SDS + triglycerides SDS + (− 1 × HDL-C SDS) + insulin SDS [5, 17]. In this score, SBP and DBP were multiplied by 0.5 to achieve a single total blood pressure value [6]. Because an increase in HDL cholesterol is considered beneficial for cardiometabolic health, the inverse of HDL cholesterol was included in the formula [6]. Higher scores indicated poorer cardiometabolic health.

### Covariates

Information regarding date of birth and sex of the children was retrieved from medical records. The socioeconomic related covariates were self-reported via questionnaires around the age of 10 years. Ethnicity was defined in accordance with the classification of Statistics Netherlands using information on the country of birth of the participants and their parents and categorized into Dutch and non-Dutch. Education level of the mother was categorized into higher education (bachelor’s degree, university) or non-higher education (no/primary education, secondary school, vocational training) [13]. Information regarding household income of parents was obtained using a questionnaire at the follow-up of 10 years and was categorized into < 2800 Euros/month or ≥ 2800 Euros/month. Furthermore, sports participation and screen time of children were assessed with questionnaires at 10 years. Sport participation was categorized into < 2 h/week or ≥ 2 h/week and screen time of the child was categorized as time watching television and/or using computers for < 2 h/day or ≥ 2 h/day.

### Statistical analyses

Multivariable linear regression models were used to analyze associations of diet quality with cardiometabolic factors. Linearity assumptions were tested using natural cubic splines, and no better non-linear fit over a linear model was found between the independent and any of the outcome variables. We analyzed all associations with stepwise adjustment for covariates in three models. Potential confounders were selected based on literature. The first model was adjusted for age, sex, ethnicity, and total energy intake of child. The second model additionally included maternal educational level, household income, child’s sports participation, and screen time. Because of its potential mediating role, BMI was separately added to a third model, except for models with body fat percentage as the outcome.

As sensitivity analyses, we repeated our analyses replacing BMI with height and weight as covariates in model 3, as for some cardiometabolic outcomes such as blood pressure, height and/or weight specifically rather than BMI may confound associations. In addition, because our FFQ has been developed for and validated in a Dutch population, we repeated our analyses restricted to children with a Dutch ethnic background. To test whether associations are different for boys and girls, we included an interaction term in model 2. Analyses were stratified when the interaction term for sex was significant (*P* < 0.05). Finally, we repeated the analyses excluding each component of the diet quality score at a time (i.e., diet score including nine components instead of ten), to examine whether one specific dietary component drove the associations. Non-response analyses of dietary data were performed to compare characteristics of children with valid dietary data (*n* = 4733) to children with missing dietary data, but who still participated in the study at 8 years (*n* = 2929) [12].

To reduce attrition bias and to prevent a decreased power [18], multiple imputation (*m* = 10 imputations) was performed based on the fully conditional specification method (predictive mean matching). For our study population of children with data on dietary intake and on at least one of the cardiometabolic outcomes (*n* = 3991), we imputed missing values of covariates (0.0–31.5%) and missing values for the remaining outcomes (0.9–32.8%). Results are presented as pooled estimates based on imputed data. Statistical significance was assumed at *P* < 0.05, two-sided. We performed all statistical analyses with IBM SPSS statistics version 25.

## Results

### Population characteristics

Table 1 presents the characteristics of the included children and their mothers for the total study population and stratified by sex. Most children had a Dutch ethnic background (67.4%), and the majority of mothers had a high educational level (63.8%). The mean ± SD diet quality score was 4.5 ± 1.2 out of 10 and was similar for boys (4.5 ± 1.2) and girls (4.6 ± 1.2). Descriptive characteristics of the cardiometabolic outcomes are presented in Table 1 and Supplementary Fig. 2. Compared to those with valid dietary data, those without dietary data more often had a higher BMI, a lower educational status, and a lower household income—as described in more detail elsewhere [12].

**Table 1:** Descriptive characteristics of study participants and their parents

	Total (*N* = 3991)	Girls (*N* = 2023)	Boys (*N* = 1968)
Parental characteristics			
Maternal education, higher (%)	63.8	64.0	63.6
Net household income, ≥ €2800/month (%)	69.6	70.6	68.6
Child characteristics			
Girls (%)	50.7	–	–
Dutch ethnicity (%)	67.4	67.5	67.3
Age at dietary assessment (y)	8.1 (8.0–8.2)	8.1 (8.0–8.2)	8.1 (8.0–8.2)
Diet quality score (score range 0–10)^a^	4.5 (± 1.2)	4.6 (± 1.2)	4.5 (± 1.2)
Total energy intake (kcal/day)	1461 (1239–1703)	1398 (1191–1612)	1537 (1306–1770)
Age at outcome measurements (years)	9.7 (9.6–9.8)	9.7 (9.6–9.8)	9.7 (9.6–9.8)
Screen time, ≥ 2 h/day (%)	51.2	46.2	56.4
Participation in sports, ≥ 2 h/day (%)	66.7	59.3	74.4
Height (cm)	141.4 (137.2–145.8)	141.2 (136.8–145.8)	141.5 (137.4–145.8)
Weight (kg)	33.6 (30.2–38.0)	33.6 (30–38.2)	33.6 (30.4–37.8)
BMI (kg/m^b^)	16.8 (15.6–18.3)	16.8 (15.5–18.5)	16.7 (15.7–18.2)
Body fat mass percentage (%)	25.2 (21.1–30.6)	27.4 (23.7–32.5)	22.6 (19.0–27.7)
Systolic blood pressure (mmHg)	102.4 (97.3–107.7)	103.0 (97.7–108.0)	102.0 (97.3–107.3)
Diastolic blood pressure (mmHg)	58.0 (54.0–62.3)	58.7 (54.3–63.0)	57.3 (53.3–61.7)
HDL cholesterol (mmol/L)	1.5 (1.3–1.7)	1.4 (1.2–1.7)	1.5 (1.3–1.7)
Triglyceride levels (mmol/L)^b^	1.0 (0.72–1.34)	0.9 (0.74–1.37)	0.9 (0.69–1.31)
Insulin levels (pmol/L)^c^	170.2 (101.70–272.20)	178.1 (109.05–289.55)	162.5 (96.21–256.80)

Descriptive statistics are presented as percentages, for categorical variables mean [± standard deviation (SD)] for continuous variables with a normal distribution and medians [interquartile range (IQR)] for continuous variables with a skewed distribution

^a^Score range 0–10 on a continuous scale, with a higher score reflecting better adherence to dietary guidelines

^b^*N* = 2777, 1397 girls, and 1380 boys

^c^*N* = 2781, 1400 girls, and 1381 boys

### Associations between diet quality score and the cardiometabolic risk factor score

Table 2 shows associations between diet quality and the cardiometabolic risk factor score based on imputed data. In model 1, a one-unit higher diet quality score was associated with a 0.14 lower cardiometabolic risk factor score (95% CI − 0.22, − 0.07). This association attenuated after adjustment for socioeconomic and lifestyle factors in model 2 [− 0.05 (95% CI − 0.13, 0.03)], but remained statistically significant after additional adjustment for BMI in model 3 [− 0.08 (95% CI − 0.15, − 0.01)].

**Table 2:** Associations of diet quality at 8 years with cardiometabolic risk factor scores at 10 years

	Cardiometabolic risk factor score *N* = 3991	Insulin (SDS) *N* = 3991	SBP (SDS) *N* = 3991	DBP (SDS) *N* = 3991	Triglycerides (SDS) *N* = 3991	HDL-C (SDS) *N* = 3991	% body fat mass (SDS) *N* = 3991
	*β* (95% CI)	*β* (95% CI)	*β* (95% CI)	*β* (95% CI)	*β* (95% CI)	*β* (95% CI)	*β* (95% CI)
Model 1 basic model	− 0.14 (− 0.22, − 0.07)	0.001 (− 0.03, 0.03)	− 0.05 (− 0.08, − 0.02)	− 0.06 (− 0.09, − 0.03)	− 0.02 (− 0.05, 0.01)	− 0.001 (− 0.04, 0.04)	− 0.07 (− 0.10, − 0.05)
Model 2 confounder-adjusted model	− 0.05 (− 0.13, 0.03)	0.02 (− 0.02, 0.05)	− 0.03 (− 0.06, − 0.002)	− 0.04 (− 0.07, − 0.02)	− 0.003 (− 0.04, 0.03)	− 0.01 (− 0.05, 0.02)	− 0.03 (− 0.05, 0.001)
Model 3 adjusted for BMI	− 0.08 (− 0.15, − 0.01)	0.01 (− 0.02, 0.04)	− 0.04 (− 0.06, − 0.01)	− 0.05 (− 0.07, − 0.02)	− 0.01 (− 0.04, 0.03)	− 0.01 (− 0.04, 0.03)	–

Estimates are regression coefficients and 95% confidence intervals (CI) from multivariable linear regression models per point higher diet score, based on imputed data

Model 1: adjusted for: age child, sex child, ethnicity child, total energy intake child

Model 2: confounder-adjusted model: additionally adjusted for, education mother, sports child, household income parents, screen time child

Model 3: additionally adjusted for BMI child

*SBP* systolic blood pressure, *DBP* diastolic blood pressure, *HDL-C* high-density lipoprotein cholesterol, *SDS* standard deviation score

### Association between the diet quality score and the individual components of the cardiometabolic risk factor score

Table 2 also presents associations between diet quality and individual components of the cardiometabolic risk factor score. After adjustment for confounders and BMI (model 3), a one-unit higher diet quality score was associated with a 0.04 SD lower systolic blood pressure (95% CI − 0.06, − 0.01 and with a 0.05 SD lower diastolic blood pressure in model 3 (95% CI − 0.07, − 0.02). In model 1, a one-unit higher diet quality score was associated with a 0.07 SD lower body fat percentage (95% CI − 0.10, − 0.05). However, this association attenuated and was no longer statistically significant after adjustment for confounders in model 2 [− 0.03 SD (95% CI − 0.05, 0.001)]. No associations were found between diet quality and insulin, triglycerides, HDL cholesterol, or LDL cholesterol in model 3 [0.01 SD (95% CI − 0.05, 0.06)].

### Additional analyses

Additional analyses in which BMI was substituted for weight and height showed similar results for all outcomes (Supplementary Table 1).

We observed a significant interaction of diet quality with sex for the total cardiometabolic risk factor score, systolic and diastolic blood pressure (all *P*-for-interaction < 0.05). Therefore, we stratified our analyses for these outcomes for sex (Supplementary Table 2). Effect estimates were in similar directions for boys and girls, but stronger among boys. For example, diet quality was associated with a 0.09 (95% CI − 0.17, − 0.01) lower cardiometabolic score among boys, but not statistically significant among girls [− 0.07 (95% CI − 0.16, 0.03)]. Similar differences were observed for systolic blood pressure [− 0.05 SD (95% CI − 0.08, − 0.01)] among boys and [− 0.03 SD (95% CI − 0.06, 0.01)] among girls and for diastolic blood pressure [− 0.06 SD (95% CI 0.10, − 0.02)] among boys and [− 0.04 SD (95% CI − 0.08, 0.01)] among girls.

Analyses restricted to children with a Dutch ethnic background (*N* = 2702) showed similar associations of diet quality with cardiometabolic health as for the total study population (Supplementary Tables 34). Complete case analyses of associations between diet quality at 8 years and cardiometabolic health outcomes at 10 years are presented in Supplementary Table 5 and demonstrate that effect estimates were similar before and after imputation of the outcomes.

Finally, sensitivity analyses in which each component of the diet quality score was excluded at a time showed similar associations as for the total diet quality score, indicating that no particular food component drove the association between the diet quality score and the cardiometabolic risk factor score (Supplementary Table 6).

## Discussion

In this study, we examined associations of diet quality with a combined cardiometabolic risk factor score and individual cardiometabolic risk factors in school-age children. Our findings suggest that higher diet quality may be associated with lower cardiometabolic risk in childhood. When we further examined the individual cardiometabolic risk factors, we observed associations of higher diet quality with lower systolic and diastolic blood pressure, and a non-significant trend for a lower body fat percentage which together may have contributed to the lower cardiometabolic risk factor score. No significant associations were found for insulin, triglycerides, or HDL cholesterol.

We additionally analyzed associations of the individual diet quality components with cardiometabolic health in children. No indications were found that one specific diet quality component drove the associations, which is in line with the previous conducted study within the Generation R cohort [5].

### Interpretation and comparisons with previous studies

Our current findings of higher diet quality with lower cardiometabolic risk in children are similar to the findings of the PANIC study, a cross-sectional study based on a Finnish cohort including 736 children aged 6 and 8 years [18]. The PANIC study reported that a higher adherence to a Finnish Children Healthy Eating Index (FCHEI) was associated with a better cardiometabolic health in boys and lower triglycerides in boys and girls [18]. This index was characterized by a higher intake of vegetables and milk and a lower intake of sugary foods. Other dietary indices used in this study were the Dietary Approaches to Stop Hypertension (DASH) Score, Baltic Sea Diet Score (BSDS), and Mediterranean Diet Score (MDS). However, no associations were found between these dietary indices and cardiometabolic health. An explanation could be that these indices were developed for adults [18]. Our findings also suggest stronger associations of a healthy diet with better cardiometabolic health in boys. In contrast, we did not find associations between higher diet quality and lower triglycerides. The latter was also not found when we previously looked at diet quality and cardiometabolic health in children at age 6 years within the Generation R cohort [5]. Different findings for boys and girls could be explained by biological differences but also by cultural context, for example different eating patterns among boys and girls, although overall diet quality did not differ within our study population [19]. Future studies should further investigate sex differences in diet with cardiometabolic health in childhood.

Furthermore, our findings for a higher diet quality and low systolic and diastolic blood pressure were in line with results from the Framingham Children’s study, a longitudinal study of children (aged 3–6 years) and their parents [20] and another cross-sectional study that examined associations of dietary patterns of children at age 7 years with individual cardiometabolic risk factors at age 10 years [21]. The latter study reported associations of a dietary pattern rich in energy-dense foods, processed meat, and low in vegetables with higher blood pressure. After adjustment for BMI associations between diet and SBP remained, but attenuated for DBP. Therefore, associations with DBP might be partly explained by BMI. In our study, associations with blood pressure were not explained by BMI. It would be interesting for future research to study the role of obesity in the association between diet quality and cardiometabolic health in children.

Hypertension and other cardiometabolic risk factors in childhood are suggested to track into adulthood, thereby influencing the risk of cardiovascular diseases [22]. Therefore, it is important to improve dietary intake already in childhood to prevent the development of chronic diseases.

### Strengths and limitations of this study

Strengths of this study are the large prospective population-based cohort-based design and the availability of information on several covariates that enabled us to adjust for potential confounding factors. In contrast to previous studies on only single nutrients or food products, our study took overall diet quality into account. This method takes into account the correlations between foods and nutrients and may give a more real-life presentation of diet, as children consume a combination of different food products and nutrients [23]. Additionally, detailed information was available regarding the cardiometabolic health outcomes, which allowed us to create a continuous cardiometabolic risk factor score. A continuous score has been proven to be less prone to errors compared to a dichotomous approach [17, 24].

A limitation of our study was that the FFQ was developed for and validated in Dutch children, but our study consisted of a multi-ethnic cohort. However, sensitivity analysis restricted to children with a Dutch ethnic background showed similar results as analysis in the whole study population; therefore, suggesting no large bias due to ethnicity. Additionally, non-response analyses of dietary data suggested that the mothers of children without dietary data more often had a higher BMI, a lower educational status, and a lower household income than those for whom we had dietary data available [12].

Furthermore, the use of non-fasting blood samples may have led to non-differential misclassification of blood lipid and insulin values and may have therefore limited our possibility to pick up a potential association with these outcomes. However, previous findings indicate that non-fasting blood samples can still predict risks of cardiometabolic health [25]. Our study population may also have been too young and healthy to pick up any differences in these outcomes. Lastly, the observational design of our study could introduce residual confounding. For instance, we used screen time as a proxy for sedentary behaviour and participation in sports as a proxy for physical activity, both reported by parents. Therefore, we may not have fully covered the physical activity levels of the children.

## Conclusions

In conclusion, we observed an association between higher diet quality and better cardiometabolic health in childhood, mainly because of a lower blood pressure.

Further studies should explore associations of diet quality in childhood with long-term cardiometabolic health, to investigate whether the observed changes in blood pressure persist into adulthood and whether high blood pressure during childhood could predict hypertension in adulthood.

## Supplementary Information

Below is the link to the electronic supplementary material.

Supplementary file1 (DOCX 107 KB)

## Data Availability

Data can be obtained on request. Requests should be directed toward the management team of the Generation R Study (secretariat.epi@erasmusmc.nl), which has a protocol for approving data requests. Because of restrictions based on privacy regulations and informed consent of the participants, data cannot be made freely available in a public repository.

## Code availability

Codes can be requested through their corresponding author.
