# Supplementary material for: Diet quality and cardiometabolic health in childhood: the Generation R Study
Source: Eur J Nutr. 2021 Sep 15;61(2):729–36. doi: 10.1007/s00394-021-02673-2 (PMC8854322; doi:10.1007/s00394-021-02673-2)
Supplement: Supplementary file 1 — Supplementary file1 (DOCX 107 KB) [file 394_2021_2673_MOESM1_ESM.docx]

**SUPPLEMENTARY INFORMATION**

**Diet quality and cardiometabolic health in childhood: The Generation R Study**
**European Journal of Nutrition**

**Noreen Z. Siddiqui, Anh N. Nguyen, Susana Santos, Trudy Voortman**

**Affiliations:**

^1^ Department of Epidemiology, Erasmus MC, University Medical Center, Rotterdam, the

Netherlands.

^2^ The Generation R Study Group, Erasmus MC, University Medical Center, Rotterdam, the

Netherlands.

^3^ Department of Pediatrics, Sophia Children's Hospital, Erasmus MC, University Medical

Center, Rotterdam, the Netherlands.

**Corresponding author:** Trudy Voortman, Department of Epidemiology, Erasmus MC

E-mail: [trudy.voortman@erasmusmc.nl](mailto:trudy.voortman@erasmusmc.nl)

**
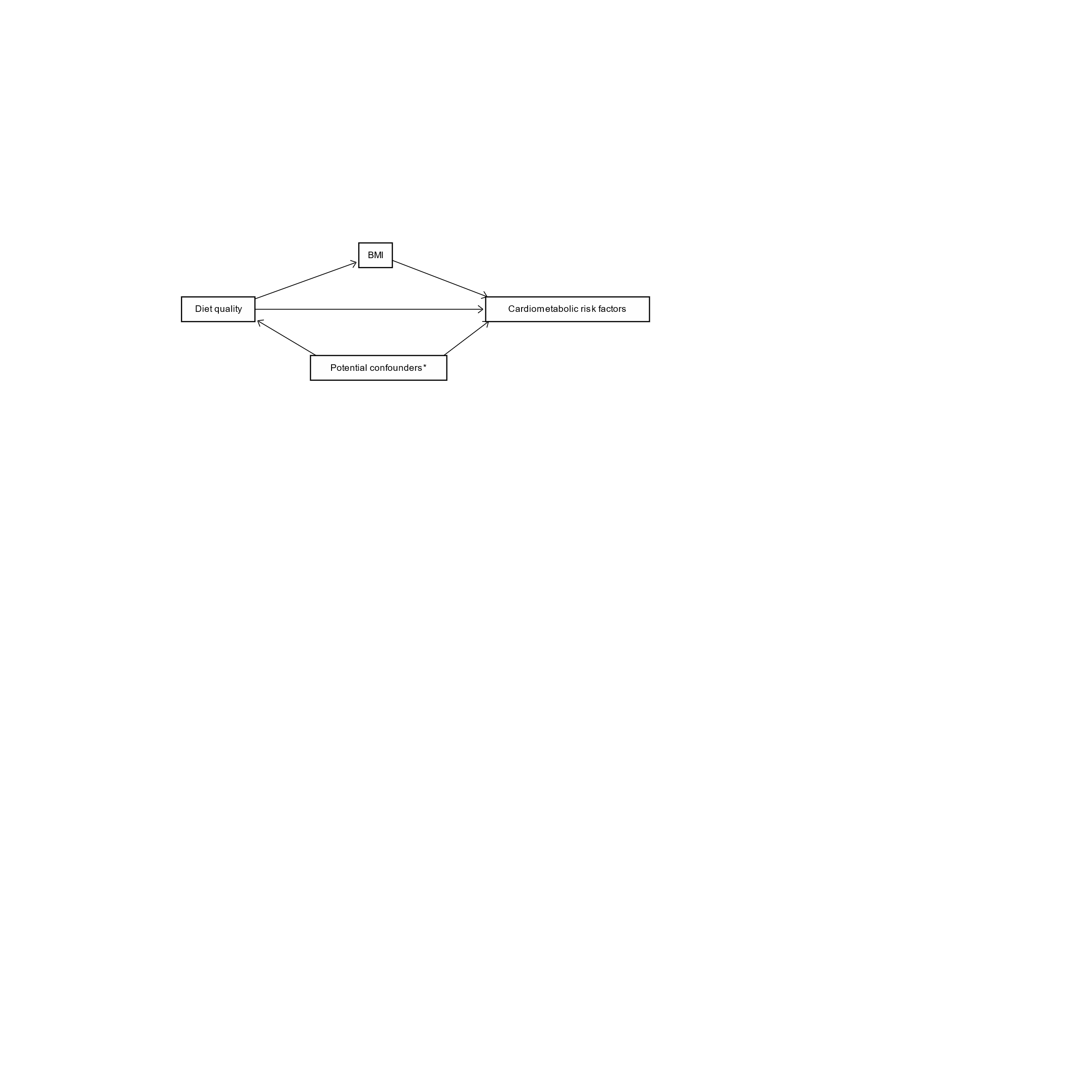
**

**Suppl. Fig. 1.** Directed Acyclic Graph (DAG) to study associations of diet quality and cardiometabolic risk factors with BMI as a potential mediator and the following *potential confounders: age child, sex child, ethnicity child, total energy intake child, education mother, sports child, household income parents, screen time child.

**
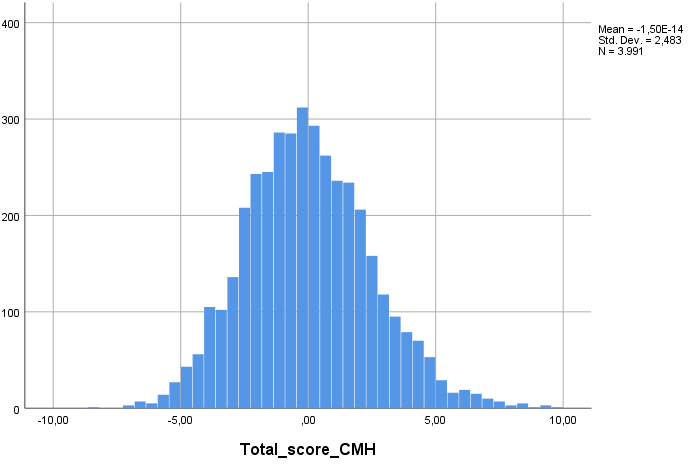
**

**Suppl. Fig. 2.** Distribution of the total cardiometabolic risk factor score

**Supplementary Table 1**Associations of diet quality at 8 years with cardiometabolic outcomes at 10 years, adjusted for weight and height (model 3)

|  | Cardiometabolic risk factor score  N = 3,991 | SBP (SDS)  N = 3,991 | DBP (SDS)  N = 3,991 |
| --- | --- | --- | --- |
|  | β (95% CI) | β (95% CI) | β (95% CI) |
| Model 1  Basic model | -0.14 (-0.22, -0.07) | -0.05 (-0.08, -0.02) | -0.06 (-0.09, -0.03) |
| Model 2  Confounder-adjusted model | -0.05 (-0.13, 0.03) | -0.03 (-0.06, -0.002) | -0.04 (-0.07, -0.02) |
| Model 3  Adjusted for weight and height | -0.08 (-0.15, -0.01) | -0.04 (-0.07, -0.02) | -0.05 (-0.08, -0.02) |
| *Estimates are regression coefficients and 95% confidence intervals (CI) from multivariable linear regression models per point higher diet score, based on imputed data.*  *Model 1: adjusted for: age child, sex child, ethnicity child, total energy intake child.  Model 2: confounder-adjusted model: additionally adjusted for, education mother, sports child, household income parents, screen time child.  Model 3: additionally adjusted for weight and height.*  *Abbreviations: SBP, systolic blood pressure; DBP, diastolic blood pressure; SDS, standard deviation score.* | | | |

**Supplementary Table 2**Associations of diet quality at 8 years with cardiometabolic risk factors scores at 10 years stratified for boys and girls

| Boys | | | | Girls | | |
| --- | --- | --- | --- | --- | --- | --- |
|  | **Cardiometabolic risk factor score**  N = 1,968 | **SBP (SDS)**  N = 1,968 | **DBP (SDS)**  N = 1,968 | **Cardiometabolic risk factor score**  N = 2,023 | **SBP (SDS)**  N = 2,023 | **DBP (SDS)**  N = 2,023 |
|  | β (95% CI) | β (95% CI) | β (95% CI) | β (95% CI) | β (95% CI) | β (95% CI) |
| Model 1  basic model | -0.20 (-0.30, -0.10) | -0.07 (-0.11, -0.04) | -0.08 (-0.12, -0.04) | -0.09 (-0.19, 0.02) | -0.03 (-0.07, 0.01) | -0.04 (-0.09, -0.01) |
| Model 2  Confounder-adjusted model | -0.10 (-0.20, -0.002) | -0.05 (-0.09, -0.01) | -0.06 (-0.10, -0.02) | -0.004 (-0.13, 0.10) | -0.01 (-0.05, 0.03) | -0.03 (-0.07, 0.01) |
| Model 3  Adjusted for BMI | -0.09 (-0.17, -0.01) | -0.05 (-0.08, -0.01) | -0.06 (-0.10, -0.02) | -0.07(-0.16, 0.03) | -0.03 (-0.06, 0.01) | -0.04 (-0.08, 0.01) |
| *Estimates are regression coefficients and 95% confidence intervals (CI) from multivariable linear regression models per point higher diet score, based on imputed data.*  *Model 1: adjusted for: age child, sex child, ethnicity child, total energy intake child.  Model 2: confounder-adjusted model: additionally adjusted for, education mother, sports child, household income parents, screen time child.  Model 3: additionally adjusted for BMI child.*  *Abbreviations: SBP, systolic blood pressure; DBP, diastolic blood pressure; SDS, standard deviation score.* | | | | | | |

**Supplementary Table 3**Associations of diet quality at 8 years with cardiometabolic outcomes at 10 years, restricted to Dutch children only

|  | Cardiometabolic risk factor score  N = 2,692 | Insulin (SDS)  N = 2,692 | SBP (SDS)  N = 2,692 | DBP (SDS)  N = 2,692 | Triglycerides (SDS)  N = 2,692 | HDL-C (SDS)  N = 2,692 | % body fat mass (SDS)  N = 2,692 |  |
| --- | --- | --- | --- | --- | --- | --- | --- | --- |
|  | β (95% CI) | β (95% CI) | β (95% CI) | β (95% CI) | β (95% CI) | β (95% CI) | β (95% CI) |  |
| Model 1  basic model | -0.15 (-0.24 -0.06) | 0.01 (-0.02, 0.05) | -0.06 (-0.09, -0.02) | -0.05 (-0.08, -0.02) | -0.02 (-0.06, 0.02) | -0.01 (-0.05, 0.04) | -0.08 (-0.11, -0.05) |  |
| Model 2  Confounder-adjusted model | -0.07 (-0.17, 0.03) | 0.02 (-0.02, 0.06) | -0.04 (-0.07, -0.001) | -0.035 (-0.07, -0.000) | -0.01 (-0.05, 0.04) | -0.01 (-0.06, 0.04) | -0.04 (-0.07, -0.01) |  |
| Model 3  Adjusted for BMI | -0.09 (-0.18, -0.01) | 0.02 (-0.02, 0.05) | -0.04 (-0.07, -0.01) | -0.04 (-0.07, -0.002) | -0.01 (-0.05, 0.03) | -0.01 (-0.06, 0.04) | - |  |
| *Estimates are regression coefficients and 95% confidence intervals (CI) from multivariable linear regression models per point higher diet score, based on imputed data.*  *Model 1: adjusted for: age child, sex child, ethnicity child, total energy intake child.  Model 2: confounder-adjusted model: additionally adjusted for, education mother, sports child, household income parents, screen time child.  Model 3: additionally adjusted for BMI child.*  *Abbreviations: SBP, systolic blood pressure; DBP, diastolic blood pressure; HDL-C, high-density lipoprotein cholesterol; SDS, standard deviation score.* | | | | | | | | |

**Supplementary Table 4**Associations of the diet quality at 8 years with cardiometabolic outcomes at 10 years, stratified for boys and girls and restricted to Dutch children only

| Boys | | | | Girls | | |
| --- | --- | --- | --- | --- | --- | --- |
|  | **Cardiometabolic risk factor score**  N = 1,326 | **SBP (SDS)**  N = 1,326 | **DBP (SDS)**  N = 1,326 | **Cardiometabolic risk factor score**  N = 1,366 | **SBP (SDS)**  N = 1,366 | **DBP (SDS)**  N = 1,366 |
|  | β (95% CI) | β (95% CI) | β (95% CI) | β (95% CI) | β (95% CI) | β (95% CI) |
| Model 1  basic model | -0.19 (-0.31, -0.07) | -0.07 (-0.12, -0.02) | -0.06 (-0.11, -0.01) | -0.11 (-0.24, 0.02) | -0.04 (-0.09, 0.01) | -0.04 (-0.10, 0.01) |
| Model 2  Confounder-adjusted model | -0.10 (-0.22, 0.02) | -0.06 (-0.10, -0.01) | -0.04 (-0.09, 0.004) | -0.04 (-0.18, 0.10) | -0.02 (-0.07, 0.03) | -0.03 (-0.08, 0.02) |
| Model 3  Adjusted for BMI | -0.10 (-0.20, 0.01) | -0.05 (-0.10, -0.01) | -0.04 (-0.09, 0.01) | -0.09 (-0.21, 0.02) | -0.03 (-0.08, 0.02) | -0.03 (-0.08, 0.02) |
| *Estimates are regression coefficients and 95% confidence intervals (CI) from multivariable linear regression models per point higher diet score, based on imputed data.*  *Model 1: adjusted for: age child, sex child, ethnicity child, total energy intake child.  Model 2: confounder-adjusted model: additionally adjusted for, education mother, sports child, household income parents, screen time child.  Model 3: additionally adjusted for BMI child.*  *Abbreviations: SBP, systolic blood pressure; DBP, diastolic blood pressure; SDS, standard deviation score.* | | | | | | |

**Supplementary Table 5**Complete case analysis of associations of diet quality at 8 years with cardiometabolic outcomes at 10 years

|  | Cardiometabolic risk factor score  N = 2,680 | Insulin (SDS)  N = 2,781 | SBP (SDS)  N = 3,865 | DBP (SDS)  N = 3,866 | Triglycerides (SDS)  N = 2,777 | HDL-C (SDS)  N = 2,780 | % body fat mass (SDS)  N = 3,955 |  |
| --- | --- | --- | --- | --- | --- | --- | --- | --- |
|  | β (95% CI) | β (95% CI) | β (95% CI) | β (95% CI) | β (95% CI) | β (95% CI) | β (95% CI) |  |
| Model 1  basic model | -0.14 (-0.22 -0.06) | 0.01 (-0.03, 0.04) | -0.05 (-0.06, -0.03) | -0.06 (-0.09, -0.04) | -0.02 (-0.03, 0.000) | -0.004 (-0.02, 0.01) | -0.07 (-0.09, -0.04) |  |
| Model 2  Confounder-adjusted model | -0.04 (-0.12, 0.04) | 0.02 (-0.01, 0.06) | -0.03 (-0.05, 0.002) | -0.04 (-0.07, -0.02) | 0.000 (-0.03, 0.03) | -0.02 (-0.05, 0.02) | -0.02 (-0.05, 0.001) |  |
| Model 3  Adjusted for BMI | -0.07 (-0.13, -0.002) | 0.02 (-0.01, 0.05) | -0.03 (-0.06, -0.01) | -0.05 (-0.07, -0.02) | -0.004 (-0.04, 0.03) | -0.01 (-0.05, 0.02) | **-** |  |
| *Estimates are regression coefficients and 95% confidence intervals (CI) from multivariable linear regression models per point higher diet score.*  *Model 1: adjusted for: age child, sex child, ethnicity child, total energy intake child.  Model 2: confounder-adjusted model: additionally adjusted for, education mother, sports child, household income parents, screen time child.  Model 3: additionally adjusted for BMI child.*  *Abbreviations: SBP, systolic blood pressure; DBP, diastolic blood pressure; HDL-C, high-density lipoprotein cholesterol; SDS, standard deviation score.* | | | | | | | | |

**Supplementary Tables 6**
Association of the diet quality score excluding one component of the diet quality score at a time with the cardiometabolic risk factor score, systolic blood pressure and diastolic blood pressure

| Cardiometabolic risk factor score | | | | | | | |
| --- | --- | --- | --- | --- | --- | --- | --- |
|  | **Diet quality**  **excluding fruit**  N = 3,991 | **Diet quality**  **excluding vegetables**  N = 3,991 | **Diet quality**  **excluding grains**  N = 3,991 | **Diet quality**  **excluding fish**  N = 3,991 | **Diet quality excluding legumes**  N = 3,991 | **Diet quality excluding nuts**  N = 3,991 | **Diet quality excluding dairy**  N = 3,991 |
|  | β (95% CI) | β (95% CI) | β (95% CI) | β (95% CI) | β (95% CI) | β (95% CI) | β (95% CI) |
| Model 1  basic model | -0.16 (-0.24 -0.08) | -0.01 (-0.02, 0.05) | -0.14 (-0.22, -0.06) | -0.10 (-0.21, -0.04) | -0.17 (-0.35, -0.08) | -0.14 (-0.22, -0.06) | -0.17 (-0.25, -0.09) |
| Model 2  Confounder-adjusted model | -0.07 (-0.20, 0.01) | -0.04 (-0.13, 0.04) | -0.05 (-0.14, 0.03) | -0.03 (-0.12, 0.06) | -0.04 (-0.13, 0.04) | -0.04 (-0.12, 0.04) | -0.08 (-0.16, 0.003) |
| Model 3  Adjusted for BMI | -0.09 (-0.16, -0.02) | -0.08 (-0.15, -0.01) | -0.09 (-0.16, -0.02) | -0.06 (-0.13, 0.02) | -0.07 (-0.15, -0.001) | -0.07 (-0.155, -0.004) | -0.09 (-0.16, -0.02) |

|  | Diet quality excluding fats  N = 3,991 | Diet quality excluding SCB  N = 3,991 | Diet quality excluding meat  N = 3,991 |
| --- | --- | --- | --- |
|  | β (95% CI) | β (95% CI) | β (95% CI) |
| Model 1  basic model | -0.14 (-0.22 -0.07) | -0.15 (-0.23, 0.08) | -0.16 (-0.24, -0.09) |
| Model 2  Confounder-adjusted model | -0.05 (-0.13, -0.03) | -0.06 (-0.14, 0.02) | -0.06 (-0.14, 0.02) |
| Model 3  Adjusted for BMI | -0.09 (-0.16, -0.02) | 0.08 (-0.15, -0.01) | -0.10 (-0.17, -0.03) |

| Systolic blood pressure | | | | | | | | | | | |
| --- | --- | --- | --- | --- | --- | --- | --- | --- | --- | --- | --- |
|  | | **Diet quality excluding fruit**  N = 3,991 | | **Diet quality excluding vegetables**  N = 3,991 | | **Diet quality excluding grains**  N = 3,991 | **Diet quality excluding fish**  N = 3,991 | **Diet quality excluding legumes**  N = 3,991 | **Diet quality excluding nuts**  N = 3,991 | **Diet quality excluding dairy**  N = 3,991 | |
|  |  | β (95% CI) | | β (95% CI) | | β (95% CI) | β (95% CI) | β (95% CI) | β (95% CI) | β (95% CI) | |
| Model 1  basic model | | -0.06 (-0.09 -0.03) | | -0.05 (-0.08, -0.02) | | -0.05 (-0.08, -0.02) | -0.05 (-0.08, -0.02) | -0.06 (-0.09, -0.03) | -0.05 (-0.08, -0.02) | -0.06 (-0.08, -0.03) | |
| Model 2  Confounder-adjusted model | | -0.04 (-0.07, -0.01) | | -0.03 (-0.06, -0.002) | | -0.03 (-0.06, 0.06) | -0.03 (-0.06, 0.01) | -0.03 (-0.06, 0.002) | -0.03 (-0.06, 0.01) | -0.04 (-0.07, -0.01) | |
| Model 3  Adjusted for BMI | | -0.04 (-0.07, -0.01) | | -0.04 (-0.07, -0.01) | | -0.04 (-0.06, -0.01) | -0.03 (-0.06, -0.002) | -0.04 (-0.07, -0.01) | -0.03 (-0.06, -0.01) | -0.04 (-0.07, -0.01) | |
|  | **Diet quality excluding fats**  N = 3,991 | | **Diet quality excluding SCB**  N = 3,991 | | **Diet quality excluding meat**  N = 3,991 | | | | | |  |
|  | β (95% CI) | | β (95% CI) | | β (95% CI) | | | | | |  |
| Model 1  basic model | -0.06 (-0.08 -0.03) | | -0.05 (-0.08, -0.02) | | -0.05 (-0.08, -0.02) | | | | | |  |
| Model 2  Confounder-adjusted model | -0.04 (-0.06, -0.01) | | -0.03 (-0.05, 0.003) | | -0.03 (-0.06, 0.002) | | | | | |  |
| Model 3  Adjusted for BMI | -0.04 (-0.07, -0.02) | | -0.03 (-0.06, -0.003) | | -0.04 (-0.06, -0.01) | | | | | |  |
| Diastolic blood pressure | | | | | | | | | | | |
|  | | **Diet quality excluding** f**ruit**  N = 3,991 | | **Diet quality excluding vegetables**  N = 3,991 | | **Diet quality excluding grains**  N = 3,991 | **Diet quality excluding fish**  N = 3,991 | **Diet quality excluding legumes**  N = 3,991 | **Diet quality excluding nuts**  N = 3,991 | **Diet quality excluding dairy**  N = 3,991 | |
|  |  | β (95% CI) | | β (95% CI) | | β (95% CI) | β (95% CI) | β (95% CI) | β (95% CI) | β (95% CI) | |
| Model 1  basic model | | -0.07 (-0.10 -0.04) | | -0.07 (-0.09, -0.04) | | -0.06 (-0.09, -0.03) | -0.06 (-0.09, -0.03) | -0.07 (-0.10, -0.04) | -0.05 (-0.08, -0.03) | -0.06 (-0.09, -0.03) | |
| Model 2  Confounder-adjusted model | | -0.05 (-0.08, -0.02) | | -0.05 (-0.08, -0.02) | | -0.05 (-0.08, -0.02) | -0.04 (-0.08, -0.01) | -0.05 (-0.08, -0.01) | -0.04 (-0.07, -0.01) | -0.05 (-0.08, -0.02) | |
| Model 3  Adjusted for BMI | | -0.05 (-0.08, -0.02) | | -0.05 (-0.08, -0.02) | | -0.05 (-0.08, -0.02) | -0.05 (-0.08, -0.01) | -0.05 (-0.07, -0.02) | -0.04 (-0.07, -0.01) | -0.05 (-0.08, -0.02) | |
|  | | **Diet quality excluding fats**  N = 3,991 | | **Diet quality excluding SCB**  N = 3,991 | | **Diet quality excluding meat**  N = 3,991 |  |  |  |  | |
|  | | β (95% CI) | | β (95% CI) | | β (95% CI) |  |  |  |  | |
| Model 1  basic model | | -0.06 (-0.09, -0.03) | | -0.06 (-0.09, -0.03) | | -0.07 (-0.09, -0.04) |  |  |  |  | |
| Model 2  Confounder-adjusted model | | -0.04 (-0.07, -0.02) | | -0.04 (-0.07, -0.02) | | -0.05 (-0.08, -0.02) |  |  |  |  | |
| Model 3  Adjusted for BMI | | -0.05 (-0.08, -0.02) | | -0.05 (-0.07, -0.02) | | -0.05 (-0.08, -0.02) |  |  |  |  | |
| *Estimates are regression coefficients and 95% confidence intervals (CI) from multivariable linear regression models per point higher diet score, based on imputed data.*  *Model 1: adjusted for: age child, sex child, ethnicity child, total energy intake child.  Model 2: confounder-adjusted model: additionally adjusted for, education mother, sports child, household income parents, screen time child.  Model 3: additionally adjusted for BMI child.*  *Abbreviations: SCB, sugar containing beverages.* | | | | | | | | | | | |
